# Iron Status and Cause-Specific Mortality After Kidney Transplantation

**DOI:** 10.1016/j.xkme.2023.100766

**Published:** 2023-12-11

**Authors:** Joanna Sophia J. Vinke, Daan Kremer, Tim J. Knobbe, Niels Grote Beverborg, Stefan P. Berger, Stephan J.L. Bakker, Martin H. de Borst, Michele F. Eisenga

**Affiliations:** 1 Department of Nephrology, University Medical Center Groningen; Groningen, the Netherlands; 2 Department of Cardiology, University Medical Center Groningen; Groningen, the Netherlands

To the Editor:

Iron deficiency is highly prevalent after kidney transplantation. Previously, we reported that iron deficiency in kidney transplant recipients is associated with an increased risk of mortality, independent of anemia.^1^ Previous studies show a link between iron deficiency and impaired cardiac^2^, ^3^, ^4^ and immune function.^5^^,^^6^ To further explore the hypotheses that cardiovascular disease or susceptibility for infection are involved in the increased mortality risk of kidney transplant recipients with iron deficiency, we now investigated the associations of iron status parameters with posttransplant mortality due to specific causes, including cardiovascular disease, infections, and cancer.

We used data from the prospective Transplantlines Food and Nutrition Study involving kidney transplant recipients ≥1 year after transplantation. Cox proportional hazard analyses were used to assess associations of ferritin, reflecting iron storage, and transferrin saturation (TSAT), reflecting functionally available iron, with all-cause and cause-specific mortality. Analyses were adjusted for potential confounders, including age, sex, estimated glomerular filtration rate, 24-hour urinary protein excretion, time since transplantation, high-sensitive C-reactive protein, systolic blood pressure, smoking status, cardiovascular history, and presence of anemia. Because fibroblast growth factor 23 (FGF23), and potentially the processes regulating its expression and cleavage, could be involved in associations of iron deficiency with outcomes, we also adjusted for total FGF23 (model 3) and intact FGF23 (iFGF23, model 4) to assess to which extent the adjustment affected the hazard ratio (HR) for all-cause and cause-specific mortality. A detailed description of the methods is provided in the supplementary methods (Item S1).

We included 695 kidney transplant recipients (age, 53 ± 13 [SD] years; 57% males; estimated glomerular filtration rate, 52 ± 20 mL/min/1.73 m^2^; ferritin, 119 μg/L [interquartile range, 54-222]; and TSAT 25% ± 11%), of whom 234 (34%) reported anemia and 158 (23%) reported a history of cardiovascular disease. Further baseline characteristics are described in Table S1. During 5.4 (interquartile range, 4.8-6.1) years of follow-up, 148 (21%) of kidney transplant recipients died: 59 (40% of deaths) from cardiovascular causes, 41 (28%) from infections, 25 (17%) from cancer and 23 (16%) from multifactorial or unknown causes. A higher TSAT was independently associated with a lower risk of all-cause mortality (HR, 0.79; 95% confidence interval [CI], 0.66-0.94 per 10% increase; *P* = 0.008) (Fig 1; Table 1). In cause-specific analyses, a higher TSAT was not associated with the risk of death from infection or cancer, but was associated with a lower risk of cardiovascular mortality (HR, 0.70; 95% CI, 0.52-0.94 per 10% increase; *P* = 0.02). In addition, a TSAT <20% was independently associated with a higher risk of all-cause mortality (HR, 1.87; 95% CI, 1.31-2.68; *P* < 0.001) and cardiovascular mortality (HR, 1.95; 95% CI, 1.10-3.46; *P* = 0.02), but not with risk of death from infection or cancer specifically. Kidney transplant recipients with a ferritin level of <100 μg/L also had an increased risk of all-cause mortality (HR, 1.43; 95% CI, 1.03-2.00; *P* = 0.04). On adjustment for total FGF23, but not iFGF23 levels, the relations between TSAT or ferritin level and risk of overall or cardiovascular mortality lost significance (Table 1).

**Figure 1:**
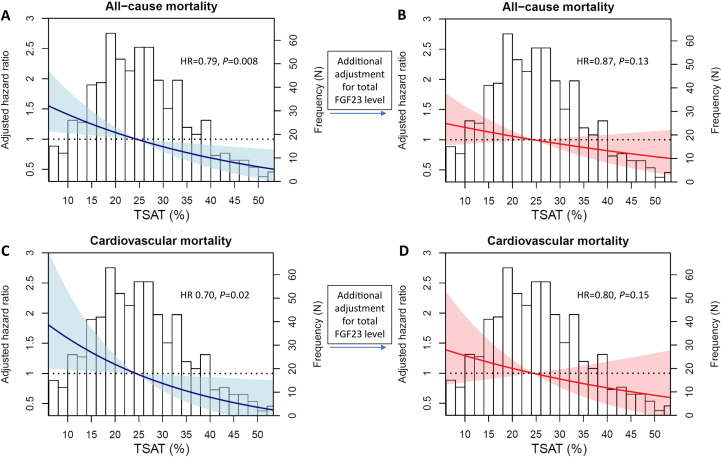
Transferrin saturation and all-cause mortality and cardiovascular mortality. (A-B) Transferrin saturation and all-cause mortality. (C-D) Transferrin saturation and cardiovascular mortality. Data were generated with a Cox proportional hazards model using restricted cubic splines. The blue and red lines represent the hazard ratio. The blue and red areas represent the 95% confidence intervals. The blue line and area represent model 2 (multivariate analysis), whereas the red line and area represent model 3 (additional adjustment for total fibroblast growth factor 23).

**Table 1:** Cox Proportional Hazards Models of the Association Between Iron Status Parameters and All-Cause or Cause-Specific Mortality

	All-Cause Mortality			Cardiovascular Mortality			Infectious Mortality			Cancer Mortality		
	Hazard Ratio (95% CI)	*P*	ΔHR	Hazard Ratio (95% CI)	*P*	ΔHR	Hazard Ratio (95% CI)	*P*	ΔHR	Hazard Ratio (95% CI)	*P*	ΔHR
**TSAT (10% increase)**												
Model 1	0.74 (0.63-0.88)	< 0.001		0.68 (0.52-0.89)	0.005		0.96 (0.72-1.29)	0.80		0.86 (0.59-1.27)	0.45	
Model 2	0.79 (0.66-0.94)	0.008		0.70 (0.52-0.94)	0.02		1.06 (0.78-1.44)	0.71		0.91 (0.60-1.38)	0.66	
Model 3	0.87 (0.72-1.04)	0.13	38%	0.80 (0.59-1.08)	0.15	33%	1.11 (0.81-1.53)	0.51	n.a.	0.96 (0.62-1.49)	0.85	n.a.
Model 4	0.79 (0.66-0.94)	0.007	0%	0.70 (0.52-0.93)	0.02	0%	1.06 (0.78-1.44)	0.71	n.a.	0.92 (0.60-1.39)	0.69	n.a.
**TSAT <20%**												
Model 1	1.91 (1.38-2.64)	< 0.001		1.94 (1.16-3.24)	0.01		1.48 (0.79-2.80)	0.23		1.65 (0.74-3.68)	0.22	
Model 2	1.87 (1.31-2.68)	< 0.001		1.95 (1.10-3.46)	0.02		1.37 (0.69-2.71)	0.37		1.82 (0.76-4.35)	0.18	
Model 3	1.60 (1.09-2.36)	0.02	31%	1.54 (0.83-2.86)	0.17	43%	1.30 (0.62-2.69)	0.49	n.a.	1.74 (0.68-4.45)	0.25	n.a.
Model 4	1.88 (1.32-2.68)	< 0.001	n.a.	1.94 (1.10-3.44)	0.02	1%	1.37 (0.69-2.71)	0.37	n.a.	1.80 (0.75-4.30)	0.19	n.a.
**Plasma ferritin**^a^												
Model 1	0.93 (0.79-1.10)	0.40		1.16 (0.89-1.51)	0.27		0.83 (0.61-1.13)	0.23		0.84 (0.57-1.25)	0.39	
Model 2	0.90 (0.76-1.06)	0.20		1.11 (0.85-1.45)	0.46		0.81 (0.60-1.11)	0.19		0.81 (0.53-1.23)	0.31	
Model 3	1.02 (0.85-1.23)	0.83	n.a.	1.36 (1.02-1.82)	0.04	n.a.	0.82 (0.59-1.16)	0.27	n.a.	0.83 (0.52-1.34)	0.45	n.a.
Model 4	0.90 (0.76-1.06)	0.21	n.a.	1.11 (0.85-1.46)	0.45	n.a.	0.81 (0.60-1.11)	0.19	n.a.	0.82 (0.53-1.25)	0.36	n.a.
**Plasma Ferritin <100 μg/L**												
Model 1	1.47 (1.06-2.03)	0.02		1.03 (0.61-1.72)	0.92		1.56 (0.84-2.89)	0.16		1.97 (0.88-4.38)	0.10	
Model 2	1.43 (1.03-2.00)	0.04		1.00 (0.59-1.71)	1.00		1.50 (0.80-2.82)	0.21		2.04 (0.90-4.64)	0.09	
Model 3	1.23 (0.86-1.75)	0.26	47%	0.78 (0.44-1.39)	0.40	n.a.	1.45 (0.75-2.80)	0.27	n.a.	1.98 (0.84-4.70)	0.12	n.a.
Model 4	1.44 (1.03-2.00)	0.03	n.a.	1.01 (0.59-1.73)	0.96	n.a.	1.50 (0.80-2.82)	0.21	n.a.	2.01 (0.88-4.58)	0.10	n.a.

*Notes:* Model 1: Crude analysis; model 2: Adjusted for age, sex, estimated glomerular filtration rate, 24-hour urinary protein excretion, time since transplantation, high-sensitive C-reactive protein, systolic blood pressure, smoking status, history of cardiovascular disease, and presence of anemia; model 3: Adjusted for model 2 + total fibroblast growth factor 23 (FGF23); Model 4: Adjusted for model 2 + intact fibroblast growth factor 23 (iFGF23) Delta. The delta hazard ratio is the percentage change in hazard ratio after additional adjustment for FGF23 (model 3) of iFGF23 (model 4).

Abbreviations: ΔHR, delta hazard ratio; n.a., not applicable; TSAT, transferrin saturation.

a ln-transformed. ΔHR Change in Hazard Ratio compared with model 2.

This study demonstrates that low TSAT levels in kidney transplant recipients are associated not only with all-cause mortality, as we previously reported,^1^^,^^7^ but with an increased risk of cardiovascular mortality specifically, independent of anemia and potential confounders. Because iron is crucial in cardiomyocyte energy metabolism and contractility,^2^ iron deficiency may impair cardiac function and thereby increase the risk of mortality. In fact, in patients with chronic heart failure, iron deficiency is associated with adverse outcomes,^3^^,^^8^ whereas iron supplementation improves ventricular function^4^ and reduces the risk of hospitalization.^9^ Furthermore, iron deficiency induces platelet aggregration^10^ and may increase thrombosis risk.^11^ Higher iron parameters may protect against incident coronary heart disease,^12^ whereas iron deficiency predicts poor outcomes after coronary artery events.^13^, ^14^, ^15^

Lower iron status was not significantly associated with an increased risk of death from infection or cancer. However, this may result from insufficient statistical power. Theoretically, because iron is pivotal for the proper functioning of the immune system,^5^^,^^6^ Iron deficiency might expose kidney transplant recipients, who are already immunocompromised by medication, to a further increased infection risk.

FGF23 explained the relationship between iron status and cardiovascular mortality to a large extent, in line with our previous study focused on all-cause mortality.^7^ Elevated levels of FGF23, a regulator of vitamin D and phosphate homeostasis, have been implicated in volume overload, left ventricular hypertrophy, and incident and worsening heart failure.^16^ We found that total FGF23, comprising both the intact form and C-terminal fragments, rather than iFGF23 alone is involved in the relation between low TSAT and outcomes. This suggests that the underlying biological processes that upregulate the production and cleavage of FGF23 or, the C-terminal fragments, are responsible for the detrimental cardiovascular effects of iron deficiency. In fact, C-terminal fragments have been found to compete with iFGF23,^17^ inhibiting phosphaturia and possibly aggravating soft tissue calcification. Our findings set the stage for prospective studies addressing the effect of iron supplementation on cardiovascular outcomes after kidney transplantation.

## Supplementary Material

Supplementary File (PDF) Item S1; Table S1.
