# Supplementary material for: Iron Status and Cause-Specific Mortality After Kidney Transplantation
Source: Kidney Med. 2023 Dec 11;6(2):100766. doi: 10.1016/j.xkme.2023.100766 (PMC10874991; doi:10.1016/j.xkme.2023.100766)
Supplement: Supplementary File (PDF) — Item S1; Table S1. [file mmc1.pdf]

Item S1. Supplementary methods

We used data from the prospective Transplantlines Food and Nutrition Study, in which baseline measurements were performed in kidney transplant receipients who were ≥1 year post-transplantation. All patients provided written informed consent. The study protocol was approved by the Medical Ethical Committee (METc 2008/186) and was in accordance with the principles of the Declaration of Helsinki. The estimated glomerular filtration rate (eGFR) was calculated using the Chronic Kidney Disease Epidemiology Collaboration (CKD-EPI) equation. Plasma iron, ferritin, and transferrin were measured using colorimetric assay, immunoassay, or immunoturbidimetric assay (Roche Diagnostics, Mannheim, Germany), respectively. Total plasma Fibroblast Growth Factor 23 (FGF23) levels were measured with human FGF23 (C-terminal) ELISA (Immutopics, Inc., San Clemente, CA), which detects both the intact hormone as well as the C-terminal cleavage products.[1] Intact plasma FGF23 levels were also measured separately with a second (sandwich C- and N-terminal) ELISA (Kainos Laboratories, Inc., Tokyo, Japan).[1] Transferrin saturation (TSAT, %) was calculated as 100 x plasma iron (μmol/L) ÷ (25 × transferrin [g/L]). For prospective analyses, we used recently updated follow-up data. Cox proportional hazards analyses were used to assess associations of ferritin, reflecting iron storage, and TSAT, reflecting functional iron status, with all-cause and cause-specific mortality. In a multivariate model (model 2), analyses were adjusted for age, sex, estimated glomerular filtration rate (eGFR), 24-hour urinary protein excretion, time since transplantation, high sensitive C-reactive protein (hs-CRP), systolic blood pressure, smoking status, cardiovascular history and presence of anemia. We then additionally adjusted for total plasma FGF23 (model 3) or intact plasma FGF23 (model 4). Percentage change in HR after adjustment for total plasma FGF23 or intact plasma FGF23 was calculated as (HR before adjustment − HR after adjustment) / (HR before adjustment − 1) × 100 to assess to which extend this attenuated – and potentially

explained - the hazard ratio (HR) for all-cause and cause-specific mortality.[2] Cardiovascular history was defined as a history of coronary artery disease, cerebrovascular accidents, peripheral arterial interventions, deep venous thrombosis or pulmonary embolism. Anemia was defined as hemoglobin <13 g/dL in men or <12 g/dL in women. Skewed variables (i.e., ferritin, 24-hour urinary protein excretion, hs-CRP, time since transplantation, total and intact FGF23) were naturally log-transformed. In none of the variables, more than 10% of the data was missing. Five datasets were multiple-imputed, and results were pooled according to Rubin's rules. In all analyses, a P-value of ≤0.05 was considered significant.

References

1. Eisenga MF, van Londen M, Leaf DE, et al. C-Terminal Fibroblast Growth Factor 23, Iron Deficiency, and Mortality in Renal Transplant Recipients. J Am Soc Nephrol. 2017 Dec;28(12):3639-3646. doi: 10.1681/ASN.2016121350.
2. Oterdoom LH, de Vries AP, van Ree RM, et al. N-terminal pro-B-type natriuretic peptide and mortality in renal transplant recipients versus the general population. Transplantation. 2009 May 27;87(10):1562-70. doi: 10.1097/TP.0b013e3181a4bb80.

Table S1 Baseline characteristics of the 695 kidney transplant recipients

**Kidney transplant recipients**	**All (N=695[a])**	**TSAT <20% (N=216)**	**TSAT ≥20% (N=475)**
**Demographics**			
Age, yrs	53 ± 13	53 ± 12	52 ± 13
Men, n (%)	395 (57)	105 (49)	289 (61)
BMI, kg/m2	27 ± 5	28 ± 5	26 ± 5
Diabetes Mellitus, n (%)	169 (24)	77 (36)	90 (19)
Previous cardiovascular disease, n (%)	158 (23)	42 (19)	115 (24)
**Transplantation Characteristics**			
History of Dialysis, n (%)	584 (84)	186 (86)	395 (83)
Time since KTx, yrs	5 (2 – 12)	4 (1 – 10)	6 (3 – 13)
Living Donor, n (%)[b]	237 (34)	71 (33)	165 (35)
**Lifestyle Parameters**			
Alcohol intake, n (%)			
- None	23 (3)	9 (4)	13 (3)
- 0-7 units/week	433 (62)	155 (72)	275 (58)
- >7 units/week	178 (26)	37 (17)	141 (30)
Smoking status, n (%)[c]			
- Never	274 (42)	77 (38)	195 (44)
- Previously	297 (45)	108 (53)	187 (42)
- Currently	84 (13)	19 (9)	65 (15)
**Laboratory parameters**			
Hemoglobin, g/dL	13.3 ± 1.7	12.7 ± 1.7	13.5 ± 1.7
Anemia, n (%)	234 (34)	91 (42)	141 (30)
Ferritin, µg/L	119 (55 – 222)	49 (27 – 126)	153 (86 – 251)
TSAT, %	25 ± 11	14 ± 4	31 ± 9
eGFR, ml/min/1.73m2	52 ± 20	51 ± 20	53 ± 20
hs-CRP, mg/L	1.6 (0.7 – 4.6)	2.5 (1.0 – 7.5)	1.3 (0.6 – 3.2)
Urine protein excretion, g/24h	0.2 (0.0 – 0.4)	0.2 (0.0 – 0.5)	0.2 (0.0 – 0.3)
Total FGF23, RU/mL	140 (95 – 232)	209 (126 – 358)	124 (89 – 179)
Intact FGF23 pg/mL	62 (43 – 99)	62 (46 – 98)	62 (42 – 100)
**Medication use**			
Proliferation inhibitors, n (%)	580 (84)	186 (86)	391 (82)
Calcineurin inhibitors, n (%)	397 (57)	141 (65)	253 (53)
Prednisolone, n (%)	689 (99)	215 (100)	470 (99)
RAAS blocker, n (%)[d]	322 (46)	92 (43)	228 (48)
Betablocker, n (%)	465 (67)	152 (70)	310 (65)
Calcium channel blocker, n (%)	185 (27)	60 (28)	124 (26)

Data are presented as mean ± Standard Deviation (SD), median with interquartile range (IQR) or number (n) with percentage (%).

Abbreviations: BMI, body mass index; hs-CRP, high-sensitive C-reactive protein; eGFR, estimated glomerular filtration rate; FGF23, fibroblast growth factor 23; RAAS, renin angiotensin aldosterone system; TSAT, transferrin saturation.

[a] TSAT available in 691 (99%).
[b] Donor type available in 693 (99%).
[c] Smoking status available in 655 (94%).
[d] RAAS blocker use available in 689 (99%).
